# Pulmonary Hygiene Protocol Reduces Incidence of Lobar Collapse in Severe Traumatic Brain Injury

**DOI:** 10.7759/cureus.12199

**Published:** 2020-12-21

**Authors:** Gerard A Baltazar, Akella Chendrasekhar, Krishna Akella, Priscilla Chow, Vladimir Rubinshteyn, Douglas Cohen, Chris Ruiz, Daniel F Genovese-Scullin, Jakey Patwari, Loren Harris

**Affiliations:** 1 Surgery, NYU Langone Hospital-Long Island, Mineola, USA; 2 Surgery, Richmond University Medical Center, Staten Island, USA; 3 Internal Medicine, Richmond University Medical Center, Staten Island, USA

**Keywords:** traumatic brain injury, pulmonary toilet, critical care, pulmonology research, ventilator-associated pneumonia, guideline directed medical therapy

## Abstract

Background

Traumatic brain injury (TBI) is a common cause of death among injured patients. In addition to neurologic sequelae which may increase mortality risk, trauma patients suffering severe TBI (Glasgow Coma Score≤8) have a predilection for pulmonary complications. We have previously demonstrated that patients with severe TBI who were intubated and mechanically ventilated are at greater risk of radiographic pulmonary lobar collapse that necessitates advanced directional suctioning and/or bronchoscopy. We sought to minimize the potentially deleterious effects of such lobar collapse by using a standardized pulmonary hygiene protocol.

Methods

We performed a retrospective comparison of lobar collapse incidence among three groups over 21 months: patients without severe TBI who were intubated and mechanically ventilated for greater than 24 hours (i.e. “NO TBI”); patients with severe TBI who were intubated and mechanically ventilated for greater than 24 hours who were not treated with a standardized pulmonary hygiene protocol (i.e. historical “CONTROL”); and patients with severe TBI who were intubated and mechanically ventilated for greater than 24 hours and who were treated with a standardized pulmonary hygiene protocol (i.e. “HYGIENE”). Our analysis excluded patients who had any significant neck injury as we had previously found that pulmonary complications are increased in this subpopulation.

Results

We reviewed the charts of 310 trauma patients (NO TBI = 104, CONTROL = 101, HYGIENE = 105) and analyzed demographics, injury severity and outcomes, including the incidence of pulmonary lobar collapse. Pulmonary hygiene protocol demonstrated a significant reduction in the incidence of lobar collapse among the HYGIENE group compared to CONTROL, approximating the incidence among patients with no TBI (11% vs 27% vs 10%, respectively, p = 0.0009). No significant difference was noted in ventilator days, intensive care unit length of stay, hospital length of stay, mortality, nor incidence of pneumonia.

Conclusion

High-risk TBI patients have a predilection towards the development of pulmonary lobar collapse, which can be significantly reduced by the use of a standardized pulmonary hygiene protocol.

## Introduction

Traumatic brain injury (TBI) is a cause of significant death and disability with a worldwide annual incidence of 10 million and steadily increasing incidence over the past decade [1-3]. Currently, 13 million individuals live with TBI-related disability in Europe and the United States (US) [4]. In the US, TBI annual incidence is 1.7 million, including 52,000 mortalities [5] and an annual cost of approximately $76.5 billion [6].

Pulmonary complications among TBI patients may result from a reduced ability to protect airways due to decreased awareness and damage to gag reflex and cough response. Pneumonia occurs in up to 60% of patients with severe TBI (defined as Glasgow coma score [GCS]≤8) [7]. It often results from aspiration at the time of injury or as a result of mechanical ventilation. Such pneumonia develops through aspiration of stomach contents and oral flora, and decreased consciousness, dry open mouth, and micro-aspiration of secretions contribute to the incidence of ventilator-associated pneumonia (VAP) [8]. Multiple studies report that the incidence of VAP among patients with severe TBI was significantly higher than patients with the same injury severity score (ISS) but no severe TBI [7,9].

In a previous study conducted at our institution, we compared patients who were intubated for more than 24 hours with similar ISS and either severe TBI or no TBI and found that the incidence of pneumonia was significantly higher in patients with severe TBI (39.6% versus 26.9%, p=0.05). We also found that patients with severe TBI who were intubated more than 24 hours were more prone to develop lobar collapse as detected by chest radiography and which necessitated advanced directional suctioning and/or bronchoscopy (27.7 % versus 11.5 %, p=0.003) [10].

In response to this increased rate of lobar collapse, we developed a standardized approach to pulmonary management in these patients. After this approach was instituted, we noticed a decrease in the incidence of lobar collapse among our patients with severe TBI. Some authors have assessed the effect of pulmonary hygiene on the incidence of VAP in patients with severe TBI [11]; however, data on the incidence and prevention of lobar collapse in intubated patients with severe TBI is lacking. We hypothesized that by using a standardized pulmonary hygiene protocol, we would decrease the incidence of lobar collapse and related outcomes.

## Materials and methods

We queried the trauma registry at our American College of Surgeons (ACS)-verified adult urban level 1 trauma centre from January 2014 to December 2017. We extracted and analyzed data on all patients with severe TBI that were intubated for greater than 24 hours before (i.e. historical controls) and after the institution of our pulmonary hygiene protocol (“CONTROL” and “HYGIENE,” respectively). We also extracted and analyzed data on non-TBI patients intubated for greater than 24 hours that had ISS matched to our original group of patients with severe TBI. This group was treated during the period after we instituted our aggressive pulmonary hygiene protocol (“NO TBI”). We then performed a detailed electronic medical record (EMR) review of data on all included patients. Our analysis excluded patients who had any neck injury as we had previously found that pulmonary complications are increased in this subpopulation [6].

Data extracted included age, sex, ISS, abbreviated injury score for a head (AIS-head), the incidence of pneumonia (VAP and healthcare-associated pneumonia [HCAP]), incidence of radiographic lobar collapse, hospital length of stay (H-LOS), ICU length of stay (ICU-LOS), ventilator days and survival to hospital discharge. Our primary outcome was an incidence of radiographic lobar collapse. Secondary outcomes were incidence of VAP, HCAP, H-LOS, ICU-LOS, ventilator days and survival to hospital discharge.

We performed a retrospective comparison of 3 groups. Our protocol for prevention of lobar collapse was instituted for both intubated and non-intubated patients (Table 1). Data were analyzed using one-way analysis of variance (ANOVA) using a commercially available statistical analysis software package (JMP 14.0®). As this was a retrospective data analysis using de-identified data, we sought and obtained an exemption from our institutional review board for the study of human subjects.

**Table 1:** Pulmonary hygiene protocol for patients suffering severe traumatic brain injury.

	Directional suction catheter system	Suctioning frequency	Chest percussion (manual or mechanical) frequency	Chest radiograph frequency	Bronchodilator therapy and/or inhaled steroids
Intubated patient with cervical spine clearance	Yes. Head should be turned to facilitate access to the right and left mainstem bronchi.	Every 2 hours.	As frequently as possible.	Daily. If lobar collapse is present, chest radiograph should be repeated within the day.	If clinically indicated.
Intubated patient without cervical spine clearance	Yes. No head turning.	Every 2 hours.	As frequently as possible.	Daily. If lobar collapse is present, chest radiograph should be repeated within the day.	If clinically indicated.
Non-intubated patient	No. Nasotracheal suction should be performed.	As frequently as possible.	As frequently as possible.	Daily. If lobar collapse is present, chest radiograph should be repeated within the day.	If clinically indicated.

## Results

We analyzed a total of 300 patients (137 females, 163 males). CONTROL patients were admitted to our ICU between January 2014 and December 2015. We implemented our pulmonary hygiene protocol in January 2016, and we extracted data on HYGIENE and NO TBI patients from March 2016 to December 2017. The mean age ± standard error of the mean for the groups was significantly higher for the HYGIENE cohort compared to the other cohorts: CONTROL 47.8 ± 2.0 years; HYGIENE 56.5 ± 2.0 years; and NO TBI 49.8 ± 2.0 years; p=0.0068.

**Figure 1:**
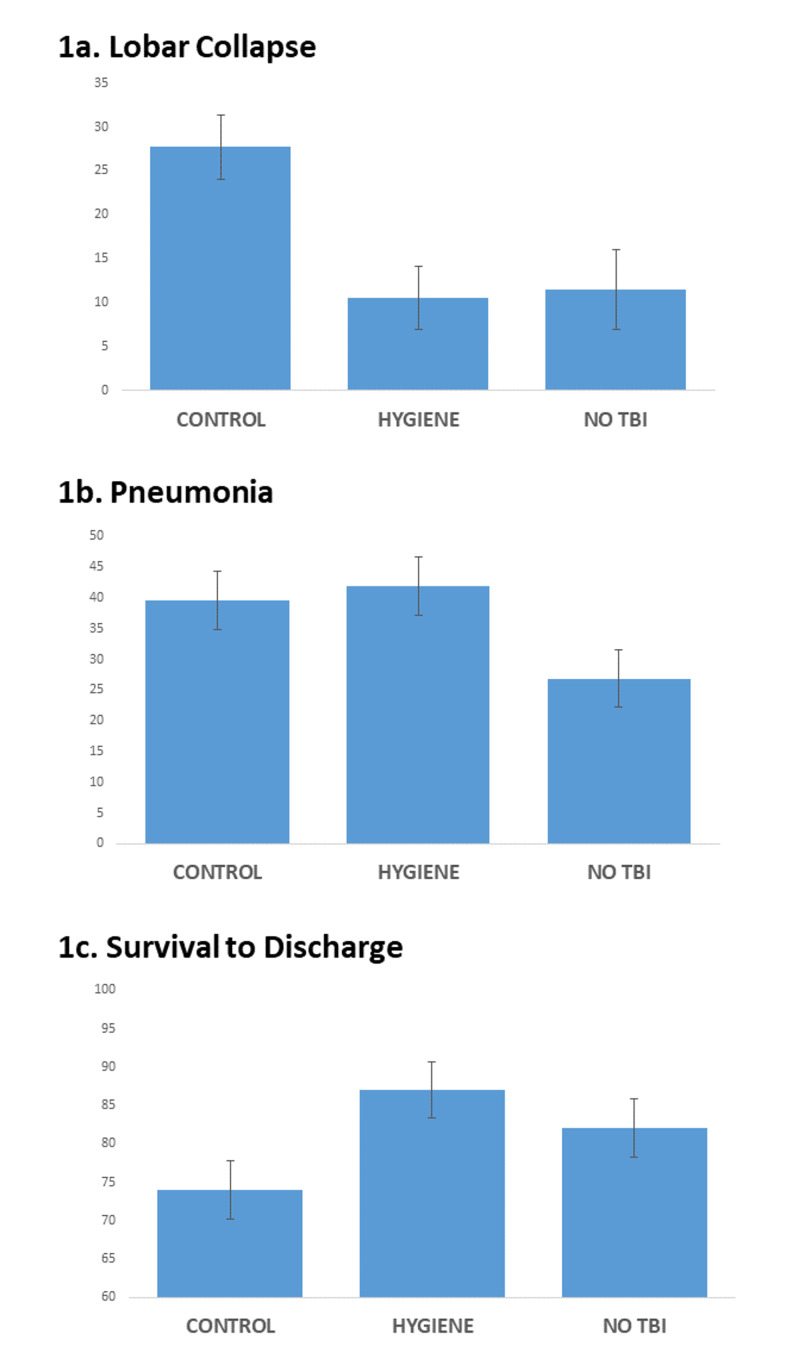
After implementation of a pulmonary hygiene protocol for patients suffering severe TBI: 1a. incidence of pulmonary lobar collapse was significantly lower than historical control and approximated incidence among injured patients without severe TBI; 1b. patients with severe TBI both before and after implementation had higher mean incidence of pneumonia compared to injured patients without severe TBI; and 1c. implementation was associated with a trend towards increased survival to discharge compared to historical control with the after implementation percent survival approximating that of ISS-matched injured patients without severe TBI. TBI- Traumatic Brain Injury; ISS- Injury Severity Score

The mean incidence of radiographic lobar collapse ± standard error of the mean was decreased in the HYGIENE cohort to levels similar to the NO TBI cohort: CONTROL 27.7 ± 3.6 %; HYGIENE 10.5 ± 3.6 %; and NO TBI 11.5 ± 4.5 %; p=0.0009 (Figure 1a).

The mean ISS ± standard error of the mean was similar among the cohorts: CONTROL 23.4 ± 1.5; HYGIENE 23.6 ± 1.4; NO TBI 20.8 ± 1.4; p=0.31. The mean AIS for a head was similar between groups with TBI: CONTROL 3.11 ± 0.039; HYGIENE 3.23 ± 0.038; NO TBI 0.106 ± 0.038. Incidence of pneumonia was significantly higher in the CONTROL and HYGIENE (i.e. severe TBI) cohorts: CONTROL 39.6 ± 4.8%; HYGIENE 41.9 ± 4.7%; NO TBI 26.9 ± 4.7%; p=0.05 (Figure 1b).

The mean H-LOS ± standard error of the mean in days was similar among the cohorts: CONTROL 16.5 ± 1.7 days; HYGIENE 14.9 ± 1.7 days; NO TBI 14.6 ± 1.7 days; p=0.70. The mean ICU-LOS ± standard error of the mean in days was also similar: CONTROL 10.0 ± 1.1 days; HYGIENE 9.8 ± 1.1 days; NO TBI 8.4 ± 1.1 days; p=0.54. The mean number of days on the ventilator ± standard error of the mean was similar: CONTROL 8.1 ± 1.0 days; HYGIENE 6.8 ± 1.0 days; NO TBI 6.6 ± 1.0 days; p=0.54. Finally, mean percent survival to hospital discharge ± standard error of the mean trended highest for the HYGIENE cohort but narrowly did not achieve statistical significance: CONTROL cohort 74 ± 3.8%; HYGIENE 87 ± 3.7%; NO TBI 82 ± 3.8%; p=0.06 (Figure 1c).

## Discussion

Severe TBI increases the risk of infection during hospitalization, including pneumonia [1-3]. Although the increased infection risk and pneumonia rates are well known [7,9], we found that in addition to the risk above factors, patients with severe TBI developed radiographic lobar collapse at a higher frequency than patients without TBI [10]. The etiology of this increased rate of lobar collapse is unclear but does not seem to be attributable to osmolar therapy use among severe TBI patients [10].

Having noted the frequency of lobar collapse, we implemented a pulmonary hygiene protocol and in the present retrospective analysis, demonstrated decreased incidence of radiographic lobar collapse but no change in the incidence of pneumonia. Other studies also note that the incidence of pneumonia is not reduced by similar pulmonary hygiene protocols [10,12]; however, the increased lobar collapse rate seen in severe TBI patients approximated ISS-matched non-TBI patients after implementation of the pulmonary hygiene protocol. This suggests that a pulmonary hygiene protocol may counteract TBI-related pathophysiologic processes associated with increased rates of lobar collapse even if rates of pneumonia and other outcomes remain unchanged.

We have continued to implement this pulmonary hygiene protocol as part of our standard of care for severe TBI patients. While LOSs and ventilator days did not demonstrate improved outcomes after implementation of the protocol, survival to hospital discharge did show a favourable trend which may not have achieved statistical significance due to several factors, including those related directly to the TBI (e.g. need for craniotomy) or statistical limitations associated with smaller sample sizes.

Although we did assess ventilator days, we did not precisely extract or analyze data on hypoxia, ventilator settings or other measures of pulmonary function which may be altered by the presence of radiographic lobar collapse. These data may benefit our understanding of any relationship between decreased rates of lobar collapse and trends towards survivability and should be included in future investigations.

In addition to the limitations above, our study suffers from the well-known limitations of retrospective study design and the limitations of generalizability of single-centre analysis. Although drawn from a reasonable time-frame (21 months), our cohort sizes were modest and may have generated statistical confounding, including sampling bias. Also, our matching process was based solely on injury severity score (ISS), and using other characteristics may have improved accuracy of the match. Finally, we did not collect data on the consistency of adherence to the pulmonary hygiene protocol. Although our results suggest enough consistency to yield an association with the improved primary outcome, future analyses should include such data in order to confirm this association.

A randomized, prospective trial analyzing patients with severe TBI would provide more clarity about the effectiveness of a pulmonary hygiene protocol, mainly if performed among multiple centres-our data demonstrate that such a trial is warranted.

## Conclusions

High-risk TBI patients have a predilection towards the development of pulmonary lobar collapse. Our study demonstrates that the implementation of a standardized pulmonary hygiene protocol for patients who have suffered a severe traumatic brain injury may significantly reduce the incidence of pulmonary lobar collapse. Our study did not detect other associated clinical outcome differences such as rates of pneumonia and survival to discharge. Examination of pulmonary hygiene protocols among larger sample sizes may demonstrate clinical outcome differences, and such studies are warranted.
